# Supplementary material for: Allergy Associated Myocardial Infarction: A Comprehensive Report of Clinical Presentation, Diagnosis and Management of Kounis Syndrome
Source: Vaccines (Basel). 2021 Dec 29;10(1):38. doi: 10.3390/vaccines10010038 (PMC8781167; doi:10.3390/vaccines10010038)
Supplement: Supplementary file 1 [file vaccines-10-00038-s001.zip › vaccines-1518222-supplementary.pdf]

Supplementary materials

Supplementary Table S1: List of manuscripts included in our review.

Manuscript Title	First Author	Publication Year	Publication Journal
Cefuroxime-induced coronary artery spasm manifesting as Kounis syndrome.	MazarakisG, et al	2005	ActaCardiologica
Penicillin allergy in cancer patients Manifesting as Kounis syndrome.	Soufras GD, et al	2005	Heart and Vessels
Hypersensitivity and Kounis syndrome due to a viper bite.	Frangides C, et al	2006	European Journal of Internal Medicine
Hymenoptera stinginduced Kounis syndrome: effects of aspirin and beta-blocker administration.	Ioannidis TI, et al	2007	International journal of Cardiology
Kounis syndrome associated with hypersensitivity to hymenoptera stings.	Kogias JS, et al	2007	International journal of Cardiology
Kounis syndrome secondary to cefuroxime-axetil use in an octogenarian.	Biteker M, et al	2008	Journal of the American geriatrics Society
Acute coronary syndrome in cisatracurium-induced anaphylacicshock: Kounis syndrome.	Yang YL, et al	2008	Official journal of the Taiwan society of anesthesiologists
Kounis syndrome secondary to amoxicillin/clavulanic acid use	Tavil Y, et al	2008	International journal of Cardiology
Serum tryptase levels in acute coronary syndromes with STelevation.	Baldomà N, et al	2009	International journal of Cardiology
Kounis syndrome: first series in Turkish patients	Biteker M, et al	2009	The Anatolian journal of Cardioloy
Acute ST-segment elevation myocardial infarction associated with diclofenac-induced anaphylaxis: case report.	De Groot JW, et al	2009	American journal of critical care
Rocuronium-induced coronary vasospasm--"Kounis syndrome".	Fagley R, et al	2009	International journal of Cardiology
Kounis Syndrome secondary to cefuroxime axetil use in an asthmatic patient.	Ilhan E, et al	2009	International journal of Cardiology
Acute ST segment elevation myocardial infarction aftersulbactam-ampicilllin induced anaphylactic shock in an adult with significant coronary artery disease:a case report	Kilic D, et al	2009	International journal of Cardiology

Kounis syndrome secondary to ibuprofen u se.	Kumar A, et al	2009	International journal of Cardiology
Acute anterior myocardial infarction after multiple bee stings. A case of Kounis syndrome.	Mytas D, et al	2009	International journal of Cardiology
Acute myocardial infarction and Kounis syndrome.	Patanè S, et al	2009	International journal of Cardiology
Kounis syndrome presenting as ST-segment elevation myocardial infarction following a hymenoptera (bee) sting.	Taggar JS, et al	2009	International journal of Cardiology
Hypersensitivity to proton pump inhibitors: lansoprazole-induced Kounis syndrome.	Vlahos N, et al	2009	International journal of Cardiology
A case of takotsubo cardiomyopathy associated with Kounis syndrome	Yanagawa Y, et al	2009	International journal of Cardiology
Cardiac arrest secondary to type 2 Kounis syndrome resulting from urticaria and angioedema.	Connor S, et al	2010	Emergency medicine journal
Kounis syndrome: report of 5 cases.	Gázquez V, et al	2010	Journal of investigational allergology and clinical immunology
Severe myocardial ischemia after concentrated epinephrine us e for the treatment of anaphylaxis: Kounis syndrome or epinephrine effect?	Izgi C, et al	2010	Heart and Lung: the journal of critical care
Acute ST elevation myocardial infarction due to allergic reaction,Kounissyndrome:A case report and review of the emergency management	Rajha E, et al	2010	The American journal of emergency medicine
Delayed Kounis syndrome and acute renal f ailure after wasp sting	Jairam A, et al	2010	International journal of Cardiology
Kounis syndrome: a manifestation of drug-eluting stent thrombosis associated with allergic reaction to contrast material.	Kogias JS, et al	2010	International journal of Cardiology
Kounis syndrome captured by coronary ang iography Computedtomography.	Park JM, et al	2010	American journal of emergency medicine
Recurrent acute myocardial infarction and Kounis syndrome.	Patanè S, et al	2010	International journal of Cardiology
Coronary vasospasm secondary to allergic reaction Followingfood ingestion: a case of type I variant Kounis syndrome	Wada T, et al	2010	Heartt and vessels
Kounis Syndrome together with Myocardial Bridging Leading to Acute Myocardial	Caglar IM, et al	2011	Case reports in medicine

Infarction at Young Age.			
Acute inferior myocardial infarction with low atrial rhythm due to propyphenazone: Kounis syndrome.	Akyel A, et al	2011	International journal of Cardiology
Kounis syndrome: two extraordinary cases.	Almpanis G, et al	2011	International journal of Cardiology
The conundrum of hypersensitivity cardiac disease: hypersensitivity myocarditis, acute hyperse nsitivity coronary syndrome (Kounis syndrome) or both?	Almpanis G, et al	2011	International journal of Cardiology
Kounis syndrome: myocardial infarction secondary to an allergic insult--a rare clinical entity.	Caglar FN, et al	2011	ActaCardiologic a
Acute coronary syndrome due to diclofenac potassium inducedanaphylaxi s: two Kounis syndrome variants in the same patient.	Cakar MA, et al	2011	The Anatolian journal of Cardiology
Oxaliplatin-induced coronary vasospasm manifesting a s Kounis syndrome: a case report.	Chang PH, et al	2011	Journal of clinical oncology
Mast cell activation disorders presenting wit h cerebral vasospasm-related symptoms: a "Kounis-like" syndrome?	González-de-Olano D, et al	2011	International journal of Cardiology
A case of coronary hypersensitivity (Kounis) syndrome associated with mid-ventricular ballooning pattern, intracoronary thrombosis and troponin elevation.	Gori T, et al	2011	International journal of Cardiology
Late stent thrombosis or Kounis syndrome?	Horjeti B, et al	2011	International journal of Cardiology
Is 5-fluorouracil-induced vasospasm a Kounis syndrome? A diagnostic challenge.	Karabay CY, et al	2011	Perfusion
Myocardial infarction following a bee sting: an example of Type IIKounis syndrome.	Karasu E, et al	2011	International journal of Cardiology
Kounis syndrome: a new twist on an old disease.	Kounis NG, et al	2011	Future cardiology
Cefuroxime-axetil induced allergic angina: an insight into classification management of Kounis syndrome.	Murat SN, et al	2011	International journal of Cardiology
Kounis syndrome in a patient with ovarian cancer and allergy to iodinated contrast media: report of a case of vasospastic angina induced by chemotherapy	Oneglia C, et al	2011	International journal of Cardiology
The Kounis-Zavras syndrome with the Samter-Beer triad.	Schwartz BG, et al	2011	Proccedings

Kounis syndrome presenting with cardiogen ic shock	Tanboğa IH, et al	2011	Journal of cardiovascular medicine
Allergic reaction reveals a non-lethal late stent thrombosis. A new subtype of Kounis syndrome?	Tsigkas G, et al	2011	International journal of Cardiology
Amoxicillin-induced Kounis syndrome manifesting as la te stentthrombosis.	Venturini E, et al	2011	International journal of Cardiology
Drug eluting stent-induced Kounis syndrome.	Venturini E, et al	2011	International journal of Cardiology
Kounis syndrome secondary to amoxicillin use in an asthmatic patient.	Viana-Tejedor A, et al	2011	International journal of Cardiology
Carboplatin-induced Kounis syndrome	Baroni M, et al	2011	Journal of cardiology cases
Two questions for Kounis syndrome: can we use magneticresonance imaging in the diagnosis and does ST elevation correlates troponin levels?	Akoz A, et al	2012	American journal of emergency medicine
Late drug eluting stent thrombosis due to acemetacine: type IIIKounis syndrome: K ounis syndrome due to acemetacine.	Akyel A, et al	2012	International journal of Cardiology
Cardiac apical aneurysm secondary to ibup rofen-induced type 1-Kounis syndrome.	Alves MF, et al	2012	International journal of Cardiology
A case of Kounis syndrome presented with sudden cardiac death.	Akyan AC, et al	2012	The Anatolian journal of Cardiology
Kounis Syndrome induced by intravenous administration of piperacillin/tazobactam: a case report	Calogiuri GF, et al	2012	International journal of Cardiology
Allergic reaction to proton pump ınhibitor: pantoprazole induced Kounis syndrome.	Canpolat U, et al	2012	International journal of Cardiology
Mast cell-related disorders presenting with Kounis syndrome.	González-de-Olano D, et al	2012	International journal of Cardiology
Allergic myocardial ischemia causing revers ible hemodynamiccollapse during gastroscopy.	Itani O, et al	2012	Anesthesia, essays and researches
Anaphylaxis from wasp stings inducing coronary thrombus.	Kasim S, et al	2012	Case reports in Cardiology
Myocardial infarction secondary to contrast agent. Contrast effect or type II Kounis syndrome?	Kocabay G, et al	2012	American journal og emergency medicine
Subclinical and clinical presentation of Kou nis syndrome: another cause of troponin elevation?	Mazarakis A, et al	2012	International journal of Cardiology
Kounis syndrome uncovers critical left main	Mazarakis A, et al	2012	International

coronary disease:<br>the question of administering epinephrine.			journal of Cardiology
ST elevation myocardial infarction with no structural lesions after a wasp sting.	Rekik S, et al	2012	The journal of emergency medicine
Type I variant of Kounis syndrome seconda ry to<br>wasp sting.	Ridolo E, et al	2012	Annals of allergy, asthma and immunology
Kounis syndrome during general anaesthes ia<br>and administration of adrenaline.	Takenaka I, et al	2012	International journal of Cardiology
[Acute inferior myocardial infarction after injection of etofenamate].	TekinYK,et al	2012	Archives of the Turkish society of Cardiology
A case of Kounis syndrome aggravated by administration of morphine.	Uluçay A, et al	2012	The Anatolian journal of Cardiology
A case of cardiac arrest with ST elevation i nduced<br>by contrast medium.	Yanagawa Y, et al	2012	American journal of emergency medicine
A case of coronary spasm with resultant ac ute<br>Myocardialinfarction: likely the result of an allergic reaction.	Yurtdaş M, et al	2012	Internal medicine
Kounis-Zavras syndrome presenting with ventricular arrhythmias and cardiogenic shock	Campo G, et al	2012	Journal of cardiology cases
Cardiogenic shock secondary to metamizole-induced type II Kounis syndrome	De Vargas, et al	2012	Revistaespaniol a de cardiologia
From treatment to diagnosis of Kounis synd rome in the catherization laboratory: the resolution of vasospastic angina after intracoronary tirofiban and nitrate therapy	Acikel S, et al	2013	International journal of Cardiology
Anisakis simplex:<br>a new etiological agent of Kounis syndrome .	Barbarroja-Escudero J, et al	2013	International journal of Cardiology
Kounis syndrome secondary to simultaneou s oral<br>amoxicillin and parenteral ampicillin use in a young man.	Bezgin T, et al	2013	Cardiovascular journal of Africa
Kounis syndrome leads to cardiogenic shock.	Chanf KL, et al	2013	Cardiovascular journal of Africa
Anaphylaxis mediated myocardial infarction in a coronary graft:<br>a new variant of Kounis syndrome (a case r eport).	Dazy K, et al	2013	International journal of Cardiology
Kounis syndrome - an atopic monster for the heart.	Gangadharan V, et al	2013	Cardiovascular diagnosis and therapy
An unusual etiology of Kounis syndrome; w arble fly	Karadeniz `m, et al	2013	Indian heart journal
Systemic mastocytosis presenting as	Lleonart R, et al	2013	Annals of

Kounis syndrome.			allergy, asthma and immunology
Intraoperative myocardial infarction: Kounis syndrome provoked by latex allergy.	Marcoux V, et al	2013	BMJ case reports
Kounis syndrome presenting as very late stent thrombosis in an everolimus-eluting stent following wasp stings.	Min JH, et al	2013	Korean circulation journal
Kounis syndrome due to angiotensin converting enzyme inhibitor.	Movva R, et al	2013	Clinical research in Cardiology
Non-<br>ST segment elevation myocardial infarction after<br>multiplebee stings. A case of "delayed" Kounis II syndrome?	Nittner-Marszalska M, et al	2013	International journal of Cardiology
A case of type I variant Kounis syndrome with Samter-Beer triad.	Prajapati JS, et al	2013	World journal of Cardiology
Peri-<br>operative cardiac arrest with ST elevation secondary to<br>gelofusin anaphylaxis - Kounis syndrome in the anaestheticroom	Shah G, et al	2013	International journal of Cardiology
Kounis syndrome resulting from anaphylaxi s to<br>diclofenac.	Tiwari AK, et al	2013	Indian journal of anesthesia
Kounis syndrome: not to be sneezed at.	Vassilioy V, et al	2013	Heart
Kounis syndrome: allergic acute coronary syndrome.	Xu M, et al	2013	Chinese medical journal
Kounis syndrome: inferior ST-segment elevation myocardial infarction following a bumblebee sting.	Zanini G, et al	2013	ActaCardiologic a
Kounis syndrome: simultaneous occurrence of<br>an allergicreaction and myocardial ischemia in a 46 year old patient after administration of<br>contrast agent.	Zlojtro M	2013	The Israerl medical association journal
Kounis syndrome triggered by diclofenac sodium injection which leads to myocardial infarction and cardiac arres	Cagliyan CE, et al	2013	Journal of Cardiology cases
Type 2 Kounis syndrome in an allergic woman: An uncommon presentation of acute coronary syndrome	Maragkoudakis S, et al	2013	Journal of cardiology cases
Drugs that may provoke Kounis Syndrome	Rodrigues M, et al	2013	Brazilian journal of anesthesiology
Kounis syndrome and acetylsalicylic acid desensitization.	Gutierrez Barrios A, et al	2014	International journal of Cardiology
Acute coronary syndrome secondary to clarithromycin: the first case and review of the literature	Bilgin M, et al	2014	Archives of the Turkish society of cardiology
Hypersensitivity myocarditis or Kounis synd rome?	Cardillo MT, et al	2014	Internal and emergency

			medicine
A Case with Repeated Recurrent Acute Cor onary Syndrome due to Pseudoephedrine Use: KounisSyndrome .	Celiker M, et al	2014	Case reports in medicine
Dangerous triplet: Polycystic ovary syndro me, oral contraceptives and Kounis syndro me.	Erol N, et al	2014	Worid journal of cardiology
Myocardial infarction secondary to morphine-induced Kounissyndrom	Akgullu C, et al	2014	Herz
Kounis syndrome, two case reports from Kr agujevac, Serbia	Davidovic G, et al	2014	American journal of cardiovascular disease
Kounis syndrome following the performanc e of skin test to amoxicillin.	González-de-Olano D, et al	2014	International journal of cardiology
An extremely rare trigger of Kounis syndro me: Actinidiachinensis.	Guler Y, et al	2014	International journal of Cardiology
Recurrent Kounis syndrome. How should be the long-term treatment of Kounis syndrome?	Günaydın ZY, et al	2014	International journal of Cardiology
Kounis syndrome manifesting as coronary aneurysm and very late coronary stent thrombosis.	Hoshi T, et al	2014	JACC cardiovascular interventions
Anaphylactic cardiovascular collapse during hemodialysis: Kounis syndrome in the dialysis room.	Mazarakis A, et al	2014	World journal of Cardiology
Kounis syndrome following cold urticaria: the swimmer's death.	Mazarakis A, et al	2014	International journal of Cardiology
Kounis syndrome after ingestion of underco oked fish: new role of intracoronary imaging techniques.	Mejía-Rentería HD, et al	2014	International journal of Cardiology
Kounis syndrome secondary to food allergy	Nachmias B, et al	2014	British journal of hospital medicine
Kounis syndrome caused by aspirin-induced asthma.	Oshima T, et al	2014	International journal of Cardiology
Acute myocardial infarction following honeybee sting.	Puttegowda B, et al	2014	BMJ case reports
A case of Kounis syndrome after a hornet s ting and literature review.	Ralapanawa DM, et al	2014	BMC research notes
Kounis syndrome: acute inferior myocardial infarction with atroventricular node block due to ceftriaxone: a first reported case.	Saleh AA, et al	2014	Annals of Saudi medicine
Intraoperative "Kounis syndrome" that improved electrocardiography changes and hemodynamic situation after administering	Sánchez VO, et al	2014	Brazilian journal of anesthesiology

nitroglycerine			
Kounis syndrome associated with brain inju ry after Hymenoptera sting: newpresentation of an established entity.	Vega F, et al	2014	International journal of Cardiology
Acute stent thrombosis due to Kounis syndrome.	Velasco E, et al	2014	International journal of Cardiology
Sudden cardiovascular collapse caused by severe anaphylaxis after cisatracurium use: a case report.	Yoon SH, et al	2014	Korean journal of anesthesiology
Acute Myocardial Infarction in Patient With Triple Negative Breast Cancer After Paclitaxel Infusion: A Case Report	Esber C, et al	2014	Cardiology research
Successful treatment of prolonged cardiopulmonary arrest of Kounis syndrome during coronary angioplasty	Akita T, et al	2015	Journal of cardiology cases
Allergic angina following wasp sting: Kounis syndrome	Anandan PK, et al	2015	Oxford medical case reports
Acute coronary syndrome due to midazolam use: Kounis syndrome during a transurethral prostatectomy.	Ateş AH, et al	2015	Archives of the Turkish society of cardiology
Brivudine induced coronary vasospasm as a manifestation of Kounis syndrome: First r eport.	Buturak A, et al	2015	International journal of Cardiology
Acute coronary syndrome and scombroid syndrome.	Cucunato M, et al	2015	International journal of Cardiology
Intravascular imaging in Kounis syndrome: role of IVUS and OCT in achieving an etiopathogenicdia gnosis.	Domínguez F, et al	2015	Cardiovascular diagnosis and therapy
Acute Lateral Myocardial Infarction Second ary to Tramadol-Induced Kounis Syndrome.	Gormel S, et al	2015	Journal of cardiothoracic and general anesthesia
Myocardial infarction associated with eosin ophilia and plasma extravasation at multiple sites. A variant of Kounis syndrome.	Gunawardena MD, et al	2015	BMJ Case reports
Kounis syndrome with Samter-Beer triad treated with intracoronary adrenaline.	Ihdayhid AR, et al	2015	Catheterization and cardiovascular interventions
Bonsai-induced Kounis Syndrome in a young male patient	Inci S, et al	2015	anatolian
Kounis syndrome: optical coherence tomography findings.	Cuesta J, et al	2015	International journal of Cardiology
Kounis syndrome: Report of 3 cases.	Katsanou K, et al	2015	International journal of Cardiology
An Extraordinary Case Associated with	L, et al	2015	Heart, lung and

an Allergic<br>Reaction to Clopidogrel: Coronary Artery Spasm or Kounis Syndrome?			circulation
Allergic acute coronary syndrome (Kounis syndrome).	Memon S, et al	2015	proccedings
Kounis syndrome	Ntuli PM, et al	2015	South African medical journal
Kounis syndrome secondary to amoxicillin/c lavulanic acid administration:<br>a case report and review of literature.	Ralapanawa DM, et al	2015	BMC research notes
Myocardial Infarction in<br>the Setting of Anaphylaxis to Celecoxib:<br>A Case of Kounis Syndrome.	Regis AC, et al	2015	The journal of emergency medicine
Kounis syndrome: a stinging case of ST-elevation myocardial infarction.	Scherbak D, et al	2015	Heart, lung and circulation
Kounis syndrome secondary to intravenous cephalosporin administration	Venkateswararao S, et al	2015	Journal of pharmacology and pharmacotherap eutics
Kounis syndrome with cardiogenic shock d uring<br>transfemoral transcatheter aortic valverepla cement.	Benedetto D, et al	2015	Coronary artery disease
A case of Kounis syndrome associated with transcatheter arterial chemoembolization for hepatocellular carcinoma	Iyonaga T, et al	2015	Journal of cardiology cases
Ventricular fibrillation by anaphylaxis following consumption of blue_skinned fish	Otsubo S, et al	2015	Acute medicine and surgery
Reversible Myocarditis and Pericarditis after Black Widow Spider Bite or Kounis Syndrome?	Yaman M, et al	2015	Case reports in cardiology
A rare cause of acute coronary syndrome: Kounis syndrome	Almeida J, et al	2016	An official journal of the Portuguese society of cardiology
Myocardial infarction following a bee sting: A case report of Kounis syndrome	Aminiahidashti H, et al	2016	Annals of cardiac anesthesia
The atopic heart: a curious case of coronary hypersensitivity	Arora S, et al	2016	The Netherlands journal of medicine
Kounis syndrome induced by cefditorenpivoxil	Barbarroja-Escudero J, et l	2016	International journal of cardiology
Kounis syndrome: Acute myocardial injury caused by multiple bee stings.	Bharadwaj P, et al	2016	Medical journal, armed forces India
Kounis syndrome triggered by a spider bite. A case report	Cervellin G, et al	2016	International journal of Cardiology
Kounis syndrome, a coronary hypersensitivity disorder: A rare case of	Cheung M, et al	2016	International journal of

amiodarone-induced coronary vasospasm and simultaneous peripheral vasodilation intraoperatively			Cardiology
Kounis syndrome following canned tuna fis h ingestion.	De Gennaro L, et al	2016	ActaclinicaBelgi ca
Kounis syndrome caused by chronic autoimmune urticaria: a case report	Erxun K, et al	2016	Journal of emergency medicine
Kounis Syndrome After Levofloxacin Intake: A Clinical Report and Cross-reactivity Study	GarcíaNúñez I, et al	2016	Journal of investigational allergology and clinical immunology
Myocardial scintigraphic evidence of Kounis syndrome: what is the aetiology of acute coronary syndrome?	Goto K, et al	2016	European heart journal
Kounis syndrome and ziprasidone	Hamera L, et al	2016	The American journal of emergency medicine
A subset of type I variant Kounis syndrome: Allergic angina syndrome and persistent presence of coronary spasm	Kim H, et al	2016	International jpurnal of Cardiology
Fatal Kounis syndrome with stent thrombosis secondary to amoxicillin/clavulanic acid use:A case report and literature review	Salouage I, et al	2016	Therapie
Kounis syndrome presenting with acute infe rior wall myocardial infarction and cardiogenic s hocks	Kesin M, et al	2016	The Anatolian journal of Cardiology
Capecitabine- induced ventricular fibrillation arrest: Possib le Kounis syndrome.	Kido K, et al	2016	Journal of oncology pharmacy practice
After Administration of Intravenous Epinephrine for bee Sting-induced Anaphylaxis: Kounis Syndrome or Epinephrine Effect?	Kounis NG, et al	2016	Chinese medical journal
Cisplatin-induced bradycardia: Cardiac toxicity or cardiac hypersensitivity and Kounis syndrome?	Kounis NG, et al	2016	International journal of Cardiology
Kounis syndrome: Identifying the trigger	Lorca R, et al	2016	International journal of Cardiology
A male patient with syncope, anaphylaxis, and ST-elevation: Hepatic and cardiac Echinococcosis presenting with Kounis syndrome	Mirijello A, et al	2016	Journal of emergency medicine
Acute extensive anterior ST elevation myocardial infarction following bee sting: a rare report of Kounis syndrome in LAD territory	Reza Karimlu M, et al	2016	Cardiovascular diagnosis and therapy
Stent dislodgement induced by	Nishi M, et al	2016	Cardiovascular

a vasodilator used for severe coronary artery spasm caused b y Kounis syndrome.			interventions and therapeutics
Successful Treatment of Kounis Syndrome Type I Presenting as Cardiac Arrest with ST Elevation	Oh KY, et al	2026	Chinese medical journal
Multiple bee stings resulting in ST elevation myocardial infarction (the Kounis syndrome)	Pelli JR, et al	2016	Proceedings
Type III Kounis syndrome after administration of an echocardiography contrast agen	Portero-Portaz JJ, et al	2026	European heart journal. Acute cardiovascular care
A Case of Kounis Type I in a Young Woman With Samter's Triad	Rayner-Hartley e, et al	2016	The Canadian journal of cardiology
Kounis syndrome associated with amoxicilli n/ clavulanic acid.	Shimi A, et al	2016	Saudi journal of anesthesia
Fluconazole-Induced Type 1 Kounis Syndrome.	Singh Mahal H, et al	2016	American journal of therapeutics
A patient with mushroom allergy; a new etiological agent of Kounis syndrome	Tepetam FM, et al	2016	Turbeculosis and thorax
Myocardial bridge as a trigger of Kounis syndrome	Venturini E, et al	2016	International journal of Cardiology
Ibuprofen-induced Kounis syndrome with diffuse ST segment depression and atrial fibrillation.	Akçay M, et al	2017	The Anatolian journal of Cardiology
Kounis Syndrome: Acute ST segment Elevation Myocardia	Antonelli D, et al	2017	The Israel medical association journal
Interesting presentation of Kounis syndrome secondary to amoxicillin/clavulanate use: coronary vasospasm and simultaneous appropriate implantable defibrillator shock	Canpolat U, et al	2017	Archives of the Turkish society of cardiology
Kounis Syndrome During Anesthesia: Presentation of Indolent Systemic Mastocytosis	De la Fuente Tornero E, et al	2017	A & A case reports
Myocardial infarction during anaphylaxis in a young healthy male with normal coronary arteries- is epinephrine the culprit?	Jayamali WD, et al	2017	BMC cardiovascular disorder
Type 1 Kounis Syndrome in Patient with Idiopathic Anaphylaxis.	Keber T, et al	2017	Case reports in cardiology
Occurrence of Kounis syndrome under anesthesia	Kerai S, et al	2017	Journal of anaesthesiology , clinical pharmacology
Death following ceftazidime-induced Kounis syndrome	Kitulwatte I, et al	2017	The medico-legal journal

An Unusual Case of Recurrent Hypersensitivity Reaction Associated with Kounis-Like Acute Coronary Syndrome.	Kundumadam S, et al	2017	Case reports in cardiology
Kounis syndrome and systemic mastocytosis in a 52-year-old man having surgery	Lerner M, et al	2017	Canadian medical association journal
Kounis syndrome: is it rare or is it underdiagnosed?	Limpo B, et al	2017	Polish heart journal
Acute anterior myocardial infarction due to stent thrombosis after mushroom consumption: a case of Kounis type III syndrome	Michas G, et al	2017	Hellenic journal of Cardiology
Management of Kounis syndrome: two case reports	Omri M, et al	2017	Journal of medical case reports
ystemicMastocytosis, Kounis Syndrome and Coronary Intervention: Case Report and Systematic Review.	Paratz ED, et al	2017	Heart, lung and circulation
Type 1 Kounis syndrome in a patient with idiopathic anaphylaxis	Sandhu M, et al	2017	Allergy and rhinology
Early stent thrombosis secondary to food allergic reaction:Kounis syndrome following rice pudding ingestion	Tzanis G, et al	2017	World journal of cardiology
Acute myocardial infarction in a patient suffering from penicillin-induced laryngeal edema: Kounis syndrome aggravated by adrenaline	Yesin M, et al	2017	Wiener klinischewochen schrift
Kounis syndrome type I in a victim of cardiopulmonary arrest after hymenoptera sting	Cholevas NV, et al	2017	Journal of cardiology cases
Wasp sting induced STEMI with complete coronary artery occlusion: a case of Kounis syndrome	Cross B, et al	2017	BMJ case reports
Kounis Syndrome Induced by Oral Intake of Diclofenac Potassium	Gunes H, et al	2017	Iranian journal of allergy, asthma and immunology
Coronary spasm secondary to cefuroxime injection, complicated with cardiogenic shock - a manifestation of Kounis syndrome: case report and literature review cefuroxime injection, complicated with cardiogenic shock – a manifestation of Kounis syndrome: case report and literature review	Mitsis A, et al	2018	European heart journal. Acute cardiovascular care
Allergic myocardial infarction (Kounis syndrome) after cefuroxime with side-chain cross-reactivity.	Absmaier M, et al	2018	The journal of allergy and clinical immunology in practice
Kounis syndrome due to hirudotherapy (leech therapy) in emergency department; a case report.	Çakmak T, et al	2018	Turkish journal of emergency medicine
Allergic recurrent coronary stent	Ferreira RM, et al	2018	Cardiovascular

thrombosis: A mini-review of Kounis syndrome.			revascularization medicine: indlucing molecular interventions
Cefuroxime-associated Kounis syndrome with unique peculiarity in perioperative prophylaxis	Gao J, et al	2018	Journal of infection and public health
Type I Kounis syndrome variant: A case report and literature review.	Haddad A, et al	2018	Avicenna journal of medicine
Timeoapismellifera and donaferens: bee sting-induced Kounis syndrome.	Katsanou K, et al	2018	Clinical medicine and laboratory medicine
Acute coronary syndrome secondary to allergic coronary vasospasm (Kounis Syndrome): a case series, follow-up and literature review	Li J, et al	2018	BMC cardiovascular disorder
Vancomycin-induced coronary artery spasm: a case of Kounis syndrome	Martinez E, et al	2018	BMJ case reports
Kounis syndrome: a paradoxal non-ST -elevation myocardialinfarction case observed after triamcinolone treatment	Yilmaz M, et al	2018	Archives of the Turkish society of cardiology
A suspected case of coronary vasospasm induced by anaphylactic shock caused by rocuronium-sugammadex complex.	Okuno A, et al	2018	Journal of clinical anesthesia
Allergic myocardial infarction following recombinant human insulin.	Özlek B, et al	2018	Heart and lung
Kounis syndrome induced by amoxicillin following vasospastic coronary event in a 22-year-old patient: a case report	Pradhan S, et al	2018	Cardiovascular diagnosis and therapy
Kounis syndrome, a disease to know: Case report and review of the literature	Sciatti E, et al	2018	Monaldi archives for chest disease
kounis syndrome induced by oral intake of aspirin case report and literature review	El Hangouche AJ, et al	2018	The Pan African medical journal
Intraprocedure type ii kounis syndrome secondary to radioiodine contrast during coronary angiography	Bhaskaran A, et al	2018	The Canadian journal of cardiology
kounis syndrome associated with selective anaphylaxis to cefazolin	Sequeira T, et al	2018	Journal og investigational allergology and clinical immunology
Perioperative Anaphylaxis Including KounisSundrome due to selective cefazolin allergy	Mota I, et al	2018	International archives of allergy and immunology
Samter-Beer triad presenting as Kounis type I variant of syndrome	Hamdi I, et al	2018	Journal of the Saudi heart association
Drug induced Kounissyndrome:does oxidative stress play a role?	Ricciardi L, et al	2018	Clinical and molecular allergy

Allergic acute coronary syndrome in exercese-induced anaplylaxis	Rosier SE, et al	2018	The Netherlands journal of medicine
Immunoglobulin E-Mediated Severe Allergy to Hyoscine Butybromide	Mendez diaz Y, et al	2018	Journal of investigational allergology and clinical immunology
Kounis syndrome induced by ranitidine	Palacios-zabalza I, et al	2018	Medicine clinica
Acute coronary stent thrombosis:a case og type 3 kounis syndrome	Tripolino C, et al	2018	Jpurnal of cardiology cases
Allergic acute coronary syndrome (kounis syndrome) in a young woman during spinal anesthesia: a case report	Vasquez LE, et al	2018	A & A practice
Kounis syndrome after rocuronium administration	Del Val Villanueva B, et al	2018	Spanish journal of ansthesiology and resuscitation
Uncommon cause of complicated myocardial infarction with normal coronary arteries in a Saudi patient	Abuosa A, et al	2018	Journal of the Saudi heart association
Vancomycin induced koynis syndrome	Leibee C, et al	2019	The American journal of emergency medicine
Anaphylactic shock versus kounis syndrome in cardiac surgery:differential diagnosis	Merino Garcia M, et al	2019	Spanish jpurnal of anesthesiology and resuscitation
Severe allergic reaction during angioplasty culminating to fatal acute stent thrombosis: An association with Kounis syndrome	Despotopoulos S, et al	2019	Heart and lung
Allergic myocardial infarction:Type I kounis syndrome following blue crab consumption	Dogan V, et al	2019	ActaclinicaBelgica
Kounis syndrome secondary to gadolinium contrast agent	Abusnina W, et l	2019	Proccedings
Kounis syndrome following solenopsis (fire ant)bite	Ng BH, et al	2019	The medical journl of Malaysia
Kounis syndrome caused by anaphylaxis without skin manifestations after cefazolin administration.	Adachi H, et al	2019	The journal of allergy and clinical immunology in practice
Kounis syndrome uncovers severe coronary disease:an unusual case of acute coronary syndrome secondary to allergic coronary vasosmasm	Conti L, et al	2019	BMJ case reports
Kounis syndrome aw first manifestation of allergic sensitization	Forlana D, et al	2019	Case reports in medicine
Electrocardiographic changes after	Jiang C, et al	2019	JAMA internal

overdose of epinephrine in a patient with anaphylaxis,Kounis syndrome or Epinephrine?			medicine
Kounissyndrome:Anaphylaxis causing coronary occlusion	Moloney N, et al	2019	Emergency medicine Australasia
Cannabis-induced acute coronary syndrome:A coincidence or not?	Landa E, et al	2019	Cureus
Angina after anaphylaxis treatment	Leow SN, et al	2019	Malaysian family physiacian: the official journal of the Academy of family physicians of Malaysia
Kounissyndrome:report of two cases with peculiar presentation and diagnostic issues	Maloberti A, et al	2019	High blood pressure & cardiovascular prevention
A case of Kounis syndrome presenting aw coronary artery spasm associated with cefazolin-induced anaphylaxis during general anesthesia	Sato M, et al	2019	JA clinical reports
Takotsubo cardiomyopathy associated with Kounissyndrome:A clinical case of the ATAK complex	Margonato D, et al	2019	Journal of cardiology cases
Gemifloxacin-induced allergic myocardial infarction:a case report	Özlek E, et al	2019	Journal of emergency medicine
A diagnostically Challenginig infusion reaction-kounis,takotsubo,or the ATAK	Mustehsan MH, et al	2019	JAMA internal medicine
An acute adverse reaction with ST elevetion induced by magnetic resonance contrstmedia:a case of kounis syndrome	Tanaka H, et al	2019	Internal medicine
Cardiogenic Shock due to kounis syndrome followin cobra bite	Priyankara WDD, et al	2019	Case reports in critical care
Acute ST-segment elevevation myocardial infarction following intramuscular diclofenac:A case of kounis syndrome	Rajh F, et al	2019	Journal of emergency medicine
A curious case of coronary vasospasm with cardiogenic shock:Type I Kounis syndrome complicated by eisiniphilic myocarditis	Ravi V, et al	2019	Cureus
Kounissyndrome:A more commonly encountered cause of acute coronary syndrome	Rodríguez-Ruiz C, et al	2019	Heart views: the official journal of the Gulf heart association
Kounis syndrome induced by contrast media:A case report and review of literature	Shibuya K, et al	2019	European journal of radiology open
Friend or foe:food-dependent exercise-induced anaphylaxis associated with acute coronarysyndrome aggravated by adrenaline and aspirin:a case report	Toya T, et al	2019	European heart journal. Case reports
Contrast media induced Kounissyndrome:A	Chien AS, et al	2019	Diagnostics

case report			
Recurrent Type III Kounis Syndrome: Will Anti-Immunoglobulin E Drug Be Another Option?	Liu Y, et al	2020	The Canadian journal of cardiology
Irreversible diffuse hypoxic-ischemic encephalopathy,secondary to type I Kounis syndrome	Anastogiannis H, et al	2020	The international journal of neuroscience
Carboplatin-induced Kounis syndrome	Tambe V, et al	2020	American journal of therapeutics
